# Dietary diversity modifies the association between household solid fuel use and sleep health in older adults

**DOI:** 10.3389/fnut.2026.1734689

**Published:** 2026-01-30

**Authors:** Xinyan Ma, Hanqing Zhao, Yan Wang, Yuhong Zhao, Daan Zhou, Minghui Sun

**Affiliations:** 1 The Third Affiliated Hospital of Jinzhou Medical University, Jinzhou, China; 2 Hunan Provincial Center for Disease Control and Prevention, Changsha, China; 3 The Fourth Affiliated Hospital of Harbin Medical University, Harbin, China; 4 Shengjing Hospital of China Medical University, Shenyang, China

**Keywords:** CLHLS, diet, dietary diversity, sleep health, solid fuels

## Abstract

**Background:**

Sleep health was crucial for healthy aging, yet it can be influenced by environmental factors and dietary habits. Evidence linking between cooking fuel use, dietary diversity, and sleep health, however, remains limited. This study aimed to investigate the aforementioned associations and to further assess the potential moderating role of dietary diversity.

**Methods:**

We included 9,121 adults aged ≥65 years from the Chinese Longitudinal Healthy Longevity Survey (CLHLS). Information on household fuel use and sleep health were collected by validated questionnaires, and dietary diversity was assessed with a simplified food frequency questionnaire. We used logistic regression models to examine the associations of solid cooking fuel use and dietary diversity with sleep health.

**Results:**

Among the 9,121 participants included in the study, 4,848 (53.15%) reported good self-reported sleep quality and 3,324 (36.44%) reported adequate sleep duration. Exposure to solid cooking fuels was associated with poor self-reported sleep quality (odds ratio [OR] = 0.86; 95% confidence interval [CI] = 0.78–0.95). In contrast, a higher dietary diversity score (DDS) was associated with better self-reported sleep quality (OR = 1.50; 95%CI = 1.37–1.64) and adequate sleep duration (OR = 1.18; 95%CI = 1.07–1.30). Similarly, a higher anti-inflammatory dietary diversity score (AIDDS) showed significant associations with better self-reported sleep quality (OR = 1.53; 95%CI = 1.39–1.67) and adequate sleep duration (OR = 1.22; 95%CI = 1.11–1.34). Notably, participants with combined exposure to clean cooking fuels and a high DDS/AIDDS had substantially greater odds of better self-reported sleep quality and adequate sleep duration than those exposed to solid fuels with a low DDS/AIDDS (*P* for interaction < 0.05).

**Conclusion:**

Our study indicates that exposure to solid cooking fuels was associated with poor self-reported sleep quality among older adults. Furthermore, higher dietary diversity may attenuate this adverse association, suggesting it is a promising target for public health interventions.

## Introduction

Sleep health is critical for psychosocial well-being and optimal physiological function (1, 2). Adults typically spend 20–40% of their day sleeping (3). Substantial epidemiological evidence indicates that sleep deficiency and disorders significantly increase the risk of cardiovascular diseases, obesity, and depression, along with other mental health conditions (4–6). It is thus imperative for public health efforts to identify modifiable factors that can improve sleep quality among older adults, particularly in light of these adverse impacts.

Household solid fuels combustion for cooking is a major source of indoor air pollution, emitting pollutants such as fine particulate matter (PM) and carbon monoxide. These pollutants degrade indoor air quality, thereby inducing systemic inflammation and oxidative stress and contributing to an estimated 4 million premature deaths annually (7, 8). In China, more than 700 million people, predominantly older adults, still rely on solid fuels for household cooking (9). Research indicates that air pollutants from such cooking practices are risk factors for coronary heart disease and increase the likelihood of non-fatal cardiovascular events (10, 11). Furthermore, a cross-sectional study of rural Chinese adults demonstrated a significantly higher prevalence of sleep disorders among solid fuel users (12). Furthermore, smaller PM particles can penetrate deep into the alveolar regions of the lungs, thereby directly interacting with pulmonary receptors and triggering systemic inflammatory responses (12, 13). Beyond the lungs, a fraction of these particles can translocate via the olfactory nerve pathway directly to the brain, constituting another pathway by which they may disrupt central nervous system functions and contribute to sleep disturbances (12, 13). These pathophysiological manifestations collectively suggest that solid cooking fuel use may impair sleep health, particularly in the elderly. However, epidemiological evidence specifically examining the relationship between exposure to different types of household solid fuels and sleep health in this population remains scarce.

Meanwhile, research interest in the relationship between dietary diversity and chronic diseases has grown. In line with previous research, dietary diversity in our study was assessed using the dietary diversity score (DDS) and the anti-inflammatory dietary diversity score (AIDDS). The DDS was designed to serve as an indicator of nutritional adequacy and a comprehensive measure of dietary patterns, as well as to capture the associated health benefits of diverse food group consumption. Evidence suggests that dietary diversity may help preserve hippocampal structure (14). A study involving 2,409 participants also reported a positive association between higher DDS and better sleep quality (15). Furthermore, several studies indicate that dietary diversity may influence sleep health in older adults through multiple pathways, such as the gut microbiota, inflammation, and oxidative stress (16–18). However, the impact of dietary diversity on sleep outcomes in the elderly remains underexplored, and it is still unclear whether dietary diversity can modify the relationship between solid fuel use and sleep health.

To investigate the potential associations between these factors and sleep health, we analyzed data from the Chinese Longitudinal Healthy Longevity Survey (CLHLS) to evaluate the relationships between household exposure to solid fuel use for cooking, adherence to dietary diversity, and sleep health in the elderly population.

## Methods

### Study population

This study included community-dwelling adults aged 65 years or older from the eighth wave of the CLHLS. The CLHLS was a nationally representative study covering most provinces in mainland China. Data on sociodemographic characteristics, lifestyle factors, and health status were collected primarily through questionnaires and physical examinations. All participants underwent face-to-face interviews conducted by trained staff. The detailed methodology of the CLHLS has been described previously (19, 20). Written informed consent was obtained from all participants prior to the survey, after the study protocol was approved by the Ethics Committee of Peking University (IRB00001052-13074).

**Figure 1:**
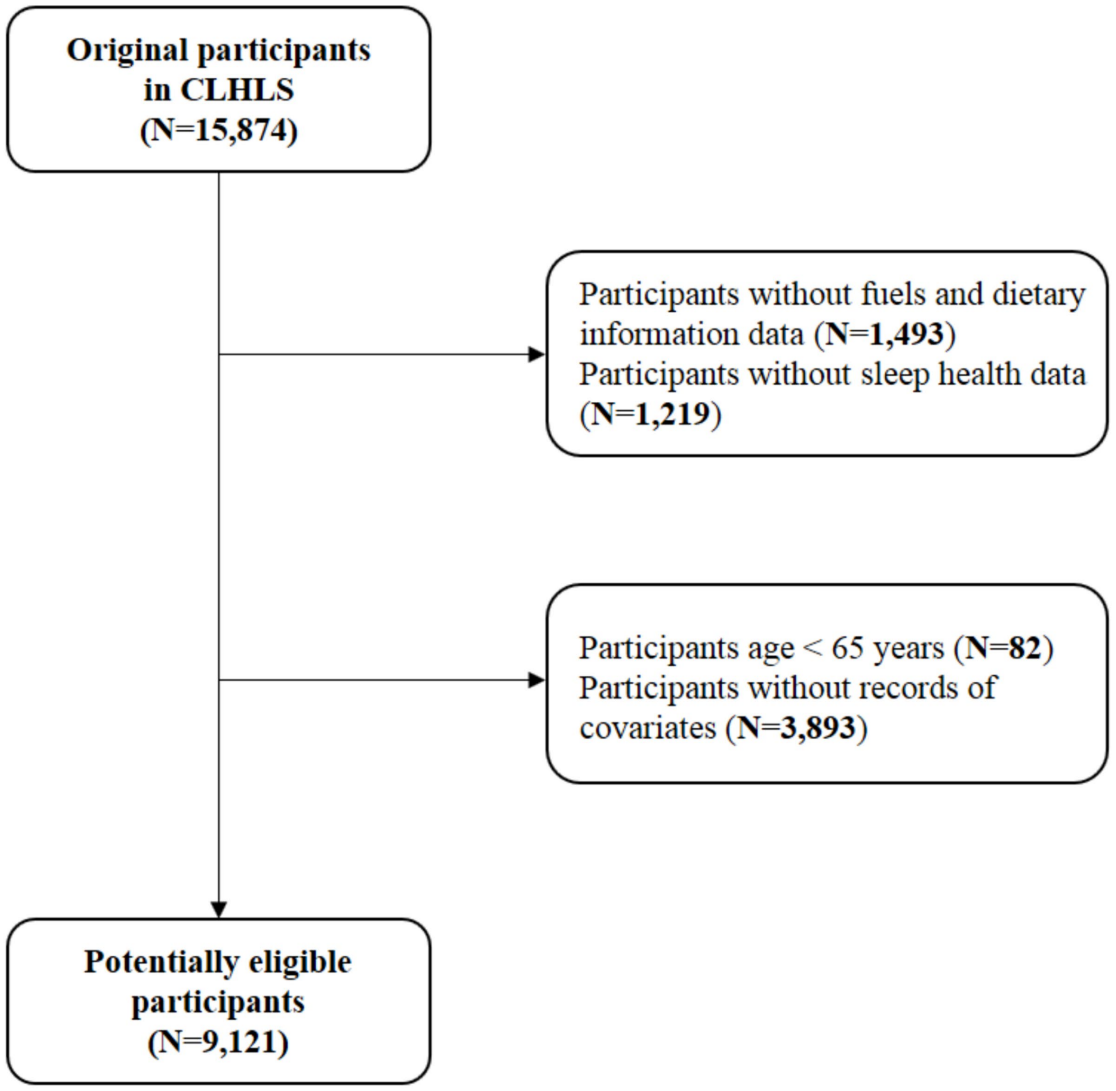
Flow chart of the study participant selection process.

A total of 15,874 participants were initially enrolled. From this study, 1,493 were excluded for missing data on fuel types or diet, 1,219 for incomplete sleep health information, 82 for age <65 years, and 3,893 for missing data on covariates, resulting in a final sample of 9,121 older adults for analysis (Figure 1).

### Assessment of household cooking fuel

Exposure to household cooking fuels was assessed via a structured questionnaire. Based on self-reported primary cooking fuel, participants were categorized as using clean fuels (natural gas, electricity, or solar energy) or solid fuels (coal or coke, firewood or straw, or charcoal) (21, 22).

### Assessment of DDS and AIDDS

During face-to-face interviews, participants’ dietary intake was assessed using a simplified food frequency questionnaire (FFQ), which has been previously validated. The question, “How often do you eat this food?” was posed for each food item (23). Information on the consumption of nine food items was collected by trained personnel. The items included fresh fruits, vegetables, legumes and legume products, nuts, meat, fish, eggs, dairy products, and tea (24). The frequency of fruit and vegetable intake was recorded as daily, often, occasionally, or seldom/almost never, with a score of 1 assigned for daily/often consumption and 0 for occasional or seldom/almost never consumption. For the remaining food items, recorded as daily, weekly, monthly, occasionally, or seldom/almost never, a score of 1 was assigned for daily/weekly intake and 0 for monthly, occasional, or seldom/almost never intake.

Dietary diversity was assessed using two primary indicators: the DDS and the AIDDS. The DDS, ranging from 0 to 9, was dichotomized into low (< 5) and high (≥ 5) diversity. The AIDDS, which evaluated the consumption of anti-inflammatory foods (vegetables, fruits, legumes and legume products, nuts, and tea), ranged from 0 to 5 and was similarly categorized as low (< 3) or high (≥ 3).

### Assessment of sleep health

Sleep health was evaluated using two questionnaire items assessing self-reported sleep quality (“How do you rate your current sleep quality?”) and sleep duration (“How many hours of sleep do you get per day?”).

Self-reported sleep quality was defined as good for responses of “very good” or “good,” and poor for “fair,” “poor,” or “very poor” (25). Sleep duration was categorized based on National Sleep Foundation recommendations for older adults (26) as short (< 7 h), optimal (7–8 h), or long (> 8 h). For analysis, it was further dichotomized into appropriate (7–8 h) and inappropriate (< 7 or > 8 h) (25).

### Assessment of covariates

Covariates were selected based on previous studies (19, 27), and included: age (continuous), gender (male/female), body mass index (BMI [continuous]), ethnicity (Han/non-Han), area of residence (urban/town/rural), annual income level (≥30,000 yuan/<30,000 yuan), marital status (cohabiting with a partner or not), exercise status (current engagement or not), alcohol consumption (current drinker or not), labor status (engaged in regular physical labor or not), smoking status (current smoker or not), hypertension (yes/no), diabetes (yes/no), and cardiovascular diseases (yes/no).

### Statistical analyses

The normality of continuous variables was assessed using the Kolmogorov–Smirnov test. Normally distributed data were presented as mean ± standard deviation and compared using the t-test; non-normally distributed data were presented as median (interquartile range) and compared using the Mann–Whitney U. Categorical variables were described as frequencies (percentages) and compared using the chi-square test. The associations between household solid cooking fuel types (including overall solid fuels, charcoal, coal/coke, and wood/crop residues) and DDS/AIDDS and sleep health were examined using multivariable logistic regression, adjusted for age, gender, ethnicity, BMI, annual income, residence area, marital status, smoking, labor status, alcohol use, exercise, hypertension, diabetes, and cardiovascular disease.

To evaluate whether dietary diversity modified the association between household solid cooking fuel use and sleep health, participants were stratified into four groups based on fuel type (divided into clean fuels and solid fuels) and DDS/AIDDS (divided into high and low). Multivariable-adjusted odds ratio (OR) with a 95% confidence interval (CI) were calculated for all exposure groups, using the group characterized by solid fuel use and low DDS/AIDDS serving as the reference. Additionally, we examined the interaction between solid cooking fuel use and DDS/AIDDS on sleep outcomes. All models controlled for a comprehensive set of demographic, lifestyle, and clinical confounders.

Subgroup analyses were conducted to assess the associations between solid cooking fuel exposure, dietary diversity (DDS/AIDDS), and sleep health across strata of age (65–79, 80–99, or ≥100 years), gender (male or female), BMI (<18.5, 18.5–23.9, or ≥24 kg/m^2^), alcohol consumption (yes or no), residence (urban, town, or rural), and smoking status (yes or no). Multiplicative interactions between solid cooking fuel and DDS/AIDDS with these stratification factors were also examined.

To evaluate the robustness of the findings, sensitivity analyses were performed: (1) further adjusting for mold exposure to account for other indoor air pollutants; (2) excluding participants with chronic respiratory diseases (bronchitis, emphysema, asthma, or pneumonia) to minimize potential bias; (3) using a more inclusive sleep duration range (6–9 h) to avoid overly restrictive definitions of sleep duration; and (4) calculating E-values to quantify the potential impact of unmeasured confounding (28). Data analysis was conducted using SAS 9.4 (SAS Institute Inc.). Statistical significance was set at a two-sided alpha level of 0.05.

## Results

### Characteristics of the study participants

As shown in Table 1, the analytical sample comprised 9,121 participants. Among them, 4,848 (53.15%) reported good sleep quality and 3,324 (36.44%) had adequate sleep duration. Participants with good sleep quality were significantly older and had a higher BMI compared to those with poor sleep quality (all *p* < 0.05). Conversely, participants with adequate sleep duration were significantly younger but also had a higher BMI than those with inadequate duration (all *p* < 0.05).

**Table 1:** Baseline characteristics according to self-reported sleep quality and sleep duration.

Characteristics	Total	Sleep quality		*p* value	Sleep duration		*p* value
		Poor	Good		Inadequate	Adequate	
Number of participants, n	9,121	4,273	4,848		5,797	3,324	
Age (years)	84.00 (74.00, 94.00)	83.00 (75.00, 93.00)	84.00 (74.00, 94.00)	0.86	85.00 (76.00, 95.00)	81.00 (72.00, 91.00)	< 0.05
BMI (kg/m^2^)	22.22 (19.60, 24.91)	22.04 (19.48, 24.73)	22.35 (19.84, 25.08)	< 0.05	22.04 (19.48, 24.73)	22.52 (20.00, 25.21)	< 0.05
Sex, n (%)				< 0.05			< 0.05
Men	4,175 (45.77)	1,730 (40.49)	2,445 (50.43)		2,523 (43.52)	1,652 (49.70)	
Women	4,946 (54.23)	2,543 (59.51)	2,403 (49.57)		3,274 (56.48)	1,672 (50.30)	
Marital status, n (%)				< 0.05			< 0.05
Live with spouse	4,034 (44.23)	1,835 (42.94)	2,199 (45.36)		2,362 (40.75)	1,672 (50.30)	
Live without spouse	5,087 (55.77)	2,438 (57.06)	2,649 (54.64)		3,435 (59.25)	1,652 (49.70)	
Race, n (%)				< 0.05			0.20
Han	8,596 (94.24)	3,994 (93.47)	4,602 (94.93)		5,447 (94.48)	3,119 (93.83)	
Other	525 (5.76)	279 (6.53)	246 (5.07)		320 (5.52)	205 (6.17)	
Residence, n (%)				0.24			< 0.05
City	2,125 (23.30)	962 (22.51)	1,163 (23.99)		1,274 (21.98)	851 (25.60)	
Town	3,088 (33.86)	1,457 (34.10)	1,631 (33.64)		1,992 (34.36)	1,096 (32.97)	
Rural	3,908 (42.85)	1,854 (43.39)	2,054 (42.37)		2,531 (43.66)	1,377 (41.43)	
Household annual income, n (%)				< 0.05			< 0.05
< 30,000 yuan	4,049 (44.39)	2,002 (46.85)	2,047 (42.22)		2,679 (46.21)	1,370 (41.22)	
≥ 30,000 yuan	5,072 (55.61)	2,271 (53.15)	2,801 (57.78)		3,118 (53.79)	1,954 (58.78)	
Smoking status, n (%)				< 0.05			< 0.05
Non-smoker	7,660 (83.98)	3,703 (86.66)	3,957 (81.62)		4,911 (84.72)	2,749 (82.70)	
Current smoker	1,461 (16.02)	570 (13.34)	891 (18.38)		886 (15.28)	575 (17.30)	
Drinking status, n (%)				0.34			< 0.05
Non-drinker	7,698 (84.40)	3,718 (87.01)	3,980 (82.10)		4,929 (85.03)	2,769 (83.30)	
Current drinker	1,423 (15.60)	555 (12.99)	868 (17.90)		868 (14.97)	555 (16.70)	
Exercise status, n (%)				< 0.05			< 0.05
No	6,081 (66.67)	3,037 (71.07)	3,044 (62.79)		4,037 (69.64)	2,044 (61.49)	
Yes	3,040 (33.33)	1,236 (28.93)	1,804 (37.21)		1,760 (30.36)	1,280 (38.51)	
Labor status, n (%)				0.65			0.06
No	2,229 (24.44)	1,035 (24.22)	1,194 (24.63)		1,379 (23.79)	850 (25.57)	
Yes	6,892 (75.56)	3,238 (75.78)	3,654 (75.37)		4,418 (76.21)	2,474 (74.43)	

BMI, body mass index. Values are numbers (percentages) for categorical variables and median (interquartile range) for continuous variables.

### Association of types of solid cooking fuels with sleep health

As summarized in Table 2, exposure to solid cooking fuels was significantly associated with poorer self-reported sleep quality compared to clean fuel use (OR = 0.86, 95% CI = 0.78–0.95). In contrast, no significant association was observed between solid fuel exposure and adequate sleep duration (OR = 0.97, 95% CI = 0.87–1.08).

**Table 2:** Association of household solid fuel use with sleep quality and sleep duration.

Variables	N _event_/N _total_	Model 1	Model 2	Model 3
		OR (95% CI)	OR (95% CI)	OR (95% CI)
Sleep quality				
Household fuel				
Clean fuels	3,533/6,464	1.0 (Reference)	1.0 (Reference)	1.0 (Reference)
Solid fuels	1,315/2,657	0.81 (0.74, 0.89)	0.81 (0.74, 0.89)	0.86 (0.78, 0.95)
*p* value		< 0.05	< 0.05	< 0.05
Sleep duration				
Household fuel				
Clean fuels	2,410/6,464	1.0 (Reference)	1.0 (Reference)	1.0 (Reference)
Solid fuels	914/2,657	0.88 (0.80, 0.97)	0.87 (0.79, 0.96)	0.97 (0.87, 1.08)
*p* value		< 0.05	< 0.05	0.54

CI, confidence interval; OR, odds ratio. Model 1: Crude model; Model 2: Adjusted for age (years), gender (men, women), and BMI (kg/m^2^); Model 3: Further adjusted for annual income level (≥30,000, <30,000 yuan), ethnicity (Han or others), exercise status (yes, no), labor status (yes, no), marital status (live with spouse, live without spouse), residence (city, town, or rural), smoking status (yes, no), drinking status (yes, no), hypertension (yes, no), diabetes (yes, no), and cardiovascular disease (yes, no).

Furthermore, analyses of specific solid fuel types revealed that only exposure to wood/straw was significantly associated with poorer self-reported sleep quality compared to clean fuels (adjusted OR [aOR] = 0.85, 95% CI = 0.76–0.94). In contrast, no significant associations were observed for coal or coke (aOR 1.05 [95% CI 0.82–1.34]) or charcoal (aOR 0.65 [95% CI 0.30–1.39]). Regarding sleep duration, no significant associations were observed for coal or coke (aOR 0.96 [95% CI 0.74–1.24]), charcoal (aOR 0.77 [95% CI 0.32–1.71]), or wood/straw (aOR 0.98 [95% CI 0.87–1.09]) when compared with clean fuel use (Supplementary Table S1).

### Association of DDS and AIDDS with sleep health

As summarized in Table 3, higher dietary diversity were consistently associated with better sleep health. Participants with a high DDS had significantly greater odds of good self-reported sleep quality (aOR = 1.50, 95% CI = 1.37–1.64) and adequate sleep duration (aOR = 1.18, 95% CI = 1.07–1.30) compared to those with a low DDS. Similarly, a high AIDDS was associated with increased odds of good self-reported sleep quality (aOR = 1.53, 95% CI = 1.39–1.67) and adequate duration (aOR = 1.22, 95% CI = 1.11–1.34). Furthermore, each one-point increase in the DDS and AIDDS was linearly associated with higher odds of both favorable sleep outcomes (sleep quality and sleep duration).

**Table 3:** Association of DDS and AIDDS with sleep quality and sleep duration.

Variables	N _event_/N _total_	Model 1	Model 2	Model 3
		OR (95% CI)	OR (95% CI)	OR (95% CI)
Sleep quality				
DDS				
Low	1,963/4,162	1.00 (Reference)	1.00 (Reference)	1.00 (Reference)
High	2,885/4,959	1.56 (1.43, 1.69)	1.54 (1.41, 1.68)	1.50 (1.37, 1.64)
*p* value		< 0.05	< 0.05	< 0.05
Continuous*	4,848/9,121	1.13 (1.11, 1.16)	1.13 (1.10, 1.15)	1.13 (1.10, 1.16)
AIDDS				
Low	2,535/5,256	1.00 (Reference)	1.00 (Reference)	1.00 (Reference)
High	2,313/3,865	1.60 (1.47, 1.74)	1.56 (1.43, 1.70)	1.53 (1.39, 1.67)
*p* value		< 0.05	< 0.05	< 0.05
Continuous*	4,848/9,121	1.25 (1.20, 1.29)	1.24 (1.19, 1.28)	1.23 (1.18, 1.28)
Sleep duration				
DDS				
Low	1,372/4,162	1.00 (Reference)	1.00 (Reference)	1.00 (Reference)
High	1,952/4,959	1.32 (1.21, 1.44)	1.26 (1.16, 1.38)	1.18 (1.07, 1.30)
*p* value		< 0.05	< 0.05	< 0.05
Continuous*		1.09 (1.07, 1.12)	1.08 (1.06, 1.10)	1.06 (1.04, 1.09)
AIDDS				
Low	1,750/5,256	1.00 (Reference)	1.00 (Reference)	1.00 (Reference)
High	1,574/3,865	1.38 (1.26, 1.50)	1.30 (1.18, 1.42)	1.22 (1.11, 1.34)
*p* value		< 0.05	< 0.05	< 0.05
Continuous*	3,324/9,121	1.17 (1.13, 1.21)	1.13 (1.09, 1.18)	1.10 (1.06, 1.15)

AIDDS, anti-inflammatory dietary diversity score; CI, confidence interval; DDS, dietary diversity score; OR, odds ratio. Model 1: Crude model; Model 2: Adjusted for age (years), gender (men, women), and BMI (kg/m^2^); Model 3: Further adjusted for annual income level (≥30,000, <30,000 yuan), ethnicity (Han or others), exercise status (yes, no), labor status (yes, no), marital status (live with spouse, live without spouse), residence (city, town, or rural), smoking status (yes, no), drinking status (yes, no), hypertension (yes, no), diabetes (yes, no), and cardiovascular disease (yes, no). *Continuous were calculated by per one score increase.

### Combined associations of solid cooking fuels use and DDS/AIDDS with sleep health

**Figure 2:**
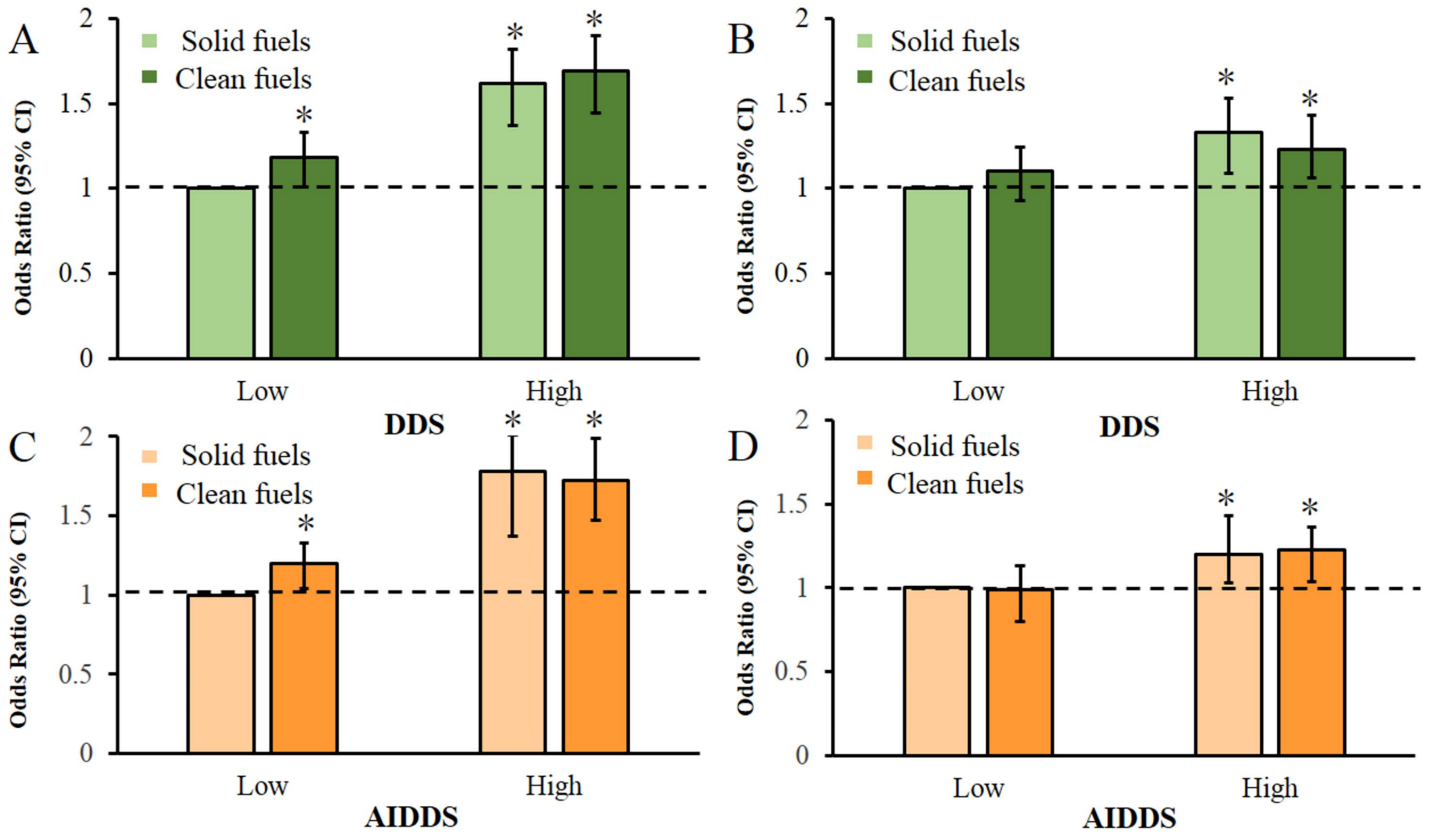
The combined effects of household fuel use and dietary diversity (as measured by DDS or AIDDS) on the odds of self-reported sleep quality (Panels **A** and **B**) and sleep duration (Panels **C** and **D**). ORs and 95% CIs were calculated using multivariable logistic regression adjusted for age (years), gender (men, women), BMI (kg/m^2^), annual income level (≥30,000, <30,000 yuan), marital status (live with spouse, live without spouse), residence (city, town, or rural), ethnicity (Han or others), exercise status (yes, no), labor status (yes, no), smoke status (yes, no), drinking status (yes, no), hypertension (yes, no), diabetes (yes, no), and cardiovascular disease (yes, no). The cutoff values were set at DDS < 5 for “low” and DDS ≥ 5 for “high” dietary diversity; for AIDDS, the corresponding cutoffs were < 3 and ≥ 3. AIDDS, anti-inflammatory dietary diversity score; CI, confidence interval; DDS, dietary diversity score. *p < 0.05.

Figure 2 illustrates the combined associations of cooking fuel exposure and dietary diversity with sleep health. Relative to the reference group (solid fuel exposure and low dietary diversity), participants with clean fuel exposure and high DDS had significantly higher odds of good self-reported sleep quality (aOR = 1.69, 95% CI = 1.48–1.94) and adequate sleep duration (aOR = 1.23, 95% CI = 1.07–1.42). Similarly, those with clean fuel exposure and high AIDDS also showed significantly increased odds for good self-reported sleep quality (aOR = 1.72, 95% CI = 1.51–1.97) and adequate duration (aOR = 1.23, 95% CI = 1.07–1.42). A significant multiplicative interaction between fuel type and DDS/AIDDS was observed for both self-reported sleep quality and sleep duration (Supplementary Table S2).

### Subgroup and sensitivity analysis

In subgroup analyses, the association between solid cooking fuel exposure and self-reported sleep quality remained significant in males, rural residents, individuals with lower BMI, and non-smokers. In contrast, no statistically significant association was observed between solid cooking fuel exposure and sleep duration in any subgroup (Supplementary Table S3). The significant association between higher DDS and better self-reported sleep quality persisted across most subgroups. In contrast, the association between DDS and adequate sleep duration was only significant in specific subgroups, including participants aged 80–99 years, males, those with normal body weight, and non-smokers (Supplementary Table S4). Additionally, the significant association between a higher AIDDS and better self-reported sleep quality persisted across all subgroups except among urban residents. In contrast, the association between a higher AIDDS and adequate sleep duration was confined to specific subgroups: participants aged 65–99 years, rural residents, those with normal body weight, and non-smokers (Supplementary Table S5).

Sensitivity analyses confirmed the robustness of the primary findings. The associations remained materially unchanged after additional adjustment for mold exposure (Supplementary Tables S6, S7) and after excluding participants with respiratory diseases, with results consistent in direction and significance with the main analysis (Supplementary Tables S8, S9). Furthermore, after re-conducting the analysis using a more inclusive sleep duration range (6–9 h), we found that the direction of the association was consistent with that of the primary analysis (Supplementary Tables S10, S11). Finally, E-value analyses suggested that the observed associations were unlikely to be fully explained by unmeasured confounding (Supplementary Table S12).

## Discussion

In this nationwide study of Chinese older adults, exposure to solid cooking fuels was significantly associated with poor self-reported sleep quality. In contrast, higher DDS/AIDDS were associated with better self-reported sleep quality and adequate sleep duration. Notably, higher dietary diversity appeared to attenuate the adverse association between solid fuel use and poor self-reported sleep quality.

Currently, evidence on the association between exposure to solid cooking fuels and sleep health remains limited. Only three studies have explored this link. For instance, a case–control study involving 1,616 Chinese participants over the 80 years of age reported an association between solid fuel use and poor sleep quality (29). A study from the China Kadoorie Biobank prospective cohort, included 283,170 rural participants and demonstrated an association between solid cooking fuel use and sleep disturbance (30). In addition, a research conducted in rural Henan, China, reported a positive association between solid cooking fuel use and poor sleep quality (12). Our findings were consistent with these studies and provide further evidence that exposure to solid cooking fuels was correlated with poor self-reported sleep quality among older adults. The underlying mechanism of this association involves exposure to particulate matter (PM)_2.5_ generated by incomplete combustion of solid fuels, which may reduce serotonergic neurotransmission and disrupt circadian rhythms, thereby contributing to daytime sleepiness and impaired nighttime sleep in the elderly (31). Furthermore, pollutants from solid fuels can impair pulmonary function, exacerbate respiratory symptoms, or induce neuroinflammation, all of which were potential pathways to sleep disturbances (32, 33). Meanwhile, pollutants from solid fuels may induce neural damage by altering blood–brain barrier integrity and promoting cortical neuron degeneration, which could ultimately impair brain function and disrupt sleep architecture. These effects were likely to be more pronounced in elderly populations experiencing age-related declines in cerebral function (29, 34). Interestingly, analyses of specific fuel types revealed that only the use of wood or straw was significantly associated with poor self-reported sleep quality compared to clean fuels (OR = 0.85, 95%CI = 0.76–0.94), whereas no significant associations were found for coal/coke or charcoal. This underscores the particularly detrimental impact of biomass fuels like wood and straw. Consequently, future public health policies should prioritize phasing out these highly polluting fuels, especially among vulnerable populations. While a full transition to clean energy remains the ultimate goal, implementing feasible interim solutions-such as improving ventilation and promoting efficient cookstoves-was of more immediate practical significance. It should be noted that the non-significant findings for coal/coke and charcoal should be interpreted with caution due to the small sample size (*N* < 300), which limited the statistical power and increases the possibility of chance findings. Therefore, large-scale, multi-center prospective studies were warranted to confirm these relationships.

Consistent with the only published study to date (15), our study also identified an association between a higher DDS and better self-reported sleep quality and adequate sleep duration. Furthermore, we also reported a significant link between a higher AIDDS and better self-reported sleep quality and adequate sleep duration among older adults. These findings suggest that adopting a diverse and anti-inflammatory diet rich in fruits, vegetables, legumes, and tea may help prevent sleep disturbances in older adults. The underlying mechanism may be that such a diet provides abundant anti-inflammatory and antioxidant compounds, which can mitigate systemic inflammation and oxidative stress, thereby improving sleep (16, 17). Furthermore, dietary diversity may influence sleep through the gut microbiota, which plays a critical role in sleep regulation (18). A varied diet can promote a favorable gut microbiome by increasing the abundance of beneficial bacteria, thereby potentially supporting healthier sleep patterns (35, 36). Therefore, the association between dietary diversity and sleep health were likely underpinned by multifaceted mechanisms, potentially involving nutrient synergy, anti-inflammatory effects, gut microbiota interactions, and reduced oxidative stress. Further experimental studies were warranted to elucidate the precise biological pathways involved. Although direct evidence linking DDS/AIDDS to sleep health was limited, these scores have been consistently associated with broader health benefits. For instance, higher DDS has been linked to reduced all-cause mortality and lower incidence of type 2 diabetes in Anglo-American cohorts (37), a 5% lower risk of dementia in a UK population (38), and a lower prevalence of metabolic syndrome in Japan (39). This established role of dietary diversity in promoting overall health provides a valuable context for interpreting its potential benefits on sleep.

Our study found a null association between solid fuel use and sleep duration in both the main and subgroup analyses. One possible explanation was that sleep duration in this study was determined by the question, “How many hours do you sleep per day?” This measure does not distinguish whether the reported sleep duration includes daytime napping. Therefore, it may fail to capture potential compensatory sleep behaviors, which could contribute to the observed null association. Additionally, we observed that solid fuel use appeared to be associated with inappropriate sleep duration in model 1 and model 2, but this association disappeared in the fully adjusted model. This suggests that, after adjusting for chronic diseases, residence, lifestyle, and occupation, the association between solid fuel use and sleep duration appears to be mediated through related determinants like the need to wake up early for strenuous work. Any remaining direct effect of solid fuel use may be too weak to significantly alter total sleep duration. Thus, the adverse impacts of solid fuel use may manifest more precisely in sleep quality than in sleep duration. Further research is necessary to confirm these findings and to inform more targeted public health interventions.

Notably, our joint-effect analysis revealed that the combination of clean cooking fuels and a higher DDS/AIDDS was associated with better self-reported sleep quality and sleep duration, and a significant interaction was detected (*P*_interaction_ < 0.05). This protective effect may be explained by the anti-inflammatory and antioxidant properties of a diverse diet (40), which could counteract the systemic inflammation and oxidative stress induced by solid fuel emissions, thereby mitigating their adverse effects on sleep (41, 42). Furthermore, a diverse diet provides an array of nutrients (e.g., vitamins C and E, minerals, polyphenols) that can directly neutralize free radicals generated from PM_2.5_ exposure, thereby alleviating oxidative damage (43, 44). Our findings demonstrate that high dietary diversity can substantially mitigate the adverse effects of solid fuel use on sleep. Therefore, promoting dietary diversity represents a viable nutritional intervention strategy to protect sleep health, especially for vulnerable populations in regions where a rapid transition to clean fuels was not immediately feasible. However, due to the inherent limitations of the study, further research on causal relationships is necessary to validate our findings.

Our study represents, to our knowledge, the first investigation into the association between solid cooking fuel exposure and sleep health and the potential moderating role of dietary diversity. Major strengths include the large, nationally representative sample and the robustness of the findings across sensitivity and subgroup analyses.

This study has several limitations. First, the cross-sectional design precludes the establishment of causal relationships between dietary diversity, household fuel use, and sleep outcomes. Furthermore, we cannot rule out the possibility of reverse causality. For instance, poor sleep patterns may themselves influence choices regarding cooking fuels or dietary habits. This potential bidirectional relationship could have affected the observed associations and must be considered when interpreting our findings. Second, the self-reported data on exposure and outcomes were susceptible to recall bias, although face-to-face interviews by trained staff may have mitigated this. Third, the use of different frequency thresholds (“often” for fruits and vegetables vs. “weekly” for other foods) to define habitual consumption in our study may affect the internal consistency and comparability of the DDS. Future research would benefit from adopting a more uniform standard for defining dietary frequency. Fourth, the findings from Chinese older adults may not be generalizable to other populations. Future longitudinal studies across diverse populations are needed. Finally, despite adjusting for numerous confounders, residual confounding (e.g., by unmeasured environmental factors) remains possible, although E-value analysis suggested that substantial unmeasured confounding would be needed to nullify the observed associations.

## Conclusion

In this national study of Chinese older adults, exposure to solid cooking fuels was associated with poor self-reported sleep quality, while higher DDS/AIDDS were associated with both better self-reported sleep quality and adequate sleep duration. Notably, high dietary diversity appeared to mitigate the adverse association between solid fuel use and sleep health. These results highlight the potential of dietary interventions and underscore the need for further research to confirm causality and elucidate mechanisms.

## Data Availability

The datasets used and/or analyzed during the current study are available from the corresponding author on reasonable request. Requests to access these datasets should be directed to Minghui Sun, sunmhcmu@163.com.
