# Supplementary material for: Dietary diversity modifies the association between household solid fuel use and sleep health in older adults
Source: Front Nutr. 2026 Jan 30;13:1734689. doi: 10.3389/fnut.2026.1734689 (PMC12903132; doi:10.3389/fnut.2026.1734689)
Supplement: Supplementary file 1 [file Table_1.DOCX]

**Supplementary Table S1.** Associations between types of household solids fuel use and sleep quality and sleep duration.

| **Variables** | **N _event_/N _total_** | **Model 1** | **Model 2** | **Model 3** |
| --- | --- | --- | --- | --- |
|  |  | ****OR (95 % CI)**** | ****OR (95 % CI)**** | ****OR (95 % CI)**** |
| **Sleep quality** |  | | | |
| **Household fuel** |  |  |  |  |
| Clean fuels | 3,533/6,464 | 1.0 (Reference) | 1.0 (Reference) | 1.0 (Reference) |
| Coal or coke | 161/299 | 0.97 (0.77, 1.22) | 1.03 (0.81, 1.31) | 1.05 (0.82, 1.34) |
| Charcoal | 12/28 | 0.62 (0.29, 1.31) | 0.61 (0.28, 1.29) | 0.65 (0.30, 1.39) |
| Firewood or straw | 1,142/2,330 | 0.80 (0.73, 0.88) | 0.79 (0.72, 0.87) | 0.85 (0.76, 0.94) |
| **Sleep duration** |  | | | |
| **Household fuel** |  |  |  |  |
| Clean fuels | 2,410/6,464 | 1.0 (Reference) | 1.0 (Reference) | 1.0 (Reference) |
| Coal or coke | 100/299 | 0.85 (0.66, 1.08) | 0.87 (0.67, 1.12) | 0.96 (0.74, 1.24) |
| Charcoal | 8/28 | 0.67 (0.28, 1.48) | 0.69 (0.29, 1.53) | 0.77 (0.32, 1.71) |
| Firewood or straw | 806/2,330 | 0.89 (0.81, 0.98) | 0.87 (0.79, 0.96) | 0.98 (0.87, 1.09) |

CI, confidence interval; OR, odds ratio.

Model 1: Crude model;

Model 2: Adjusted for age (years), gender (men, women), and BMI (kg/m^2^);

Model 3: Further adjusted for annual income level (≥30,000, <30,000 yuan), ethnicity (Han or others), exercise status (yes, no), labor status (yes, no), marital status (live with spouse, live without spouse), residence (city, town, or rural), smoking status (yes, no), drinking status (yes, no), hypertension (yes, no), diabetes (yes, no), and cardiovascular disease (yes, no).

**Supplementary Table S2.** The multiplicative interaction of solid fuels use, DDS, and AIDDS on the odds of sleep quality and sleep duration *.

|  | **Sleep quality** | **Sleep duration** |
| --- | --- | --- |
| **Multiplicative interaction** |  |  |
| *P* value |  |  |
| DDS | < 0.05 | < 0.05 |
| AIDDS | < 0.05 | 0.05 |

AIDDS, anti-inflammatory dietary diversity score**;** DDS, dietary diversity score.

* *P* value was calculated with the use of the logistic regression model with adjustment for age (years), gender (men, women), BMI (kg/m^2^), annual income level (≥30,000, <30,000 yuan), ethnicity (Han or others), exercise status (yes, no), labor status (yes, no), marital status (live with spouse, live without spouse), residence (city, town, or rural), smoking status (yes, no), drinking status (yes, no), hypertension (yes, no), diabetes (yes, no), and cardiovascular disease (yes, no).

**Supplementary Table S3.** Subgroup analyses of household solid fuel use and sleep quality and sleep duration.

|  | Sleep quality | | | Sleep duration | | |
| --- | --- | --- | --- | --- | --- | --- |
|  | **OR (95 % CI)** | *P* _value_ | *P* _interaction*_ | **OR (95 % CI)** | *P* _value_ | *P* _interaction*_ |
| Age (years) |  |  | 0.27 |  |  | 0.19 |
| 65-79 | 0.87 (0.73, 1.02) | 0.09 |  | 0.90 (0.76, 1.06) | 0.21 |  |
| 80-99 | 0.88 (0.76, 1.03) | 0.08 |  | 1.01 (0.86, 1.18) | 0.92 |  |
| ≥ 100 | 0.83 (0.63, 1.09) | 0.16 |  | 1.06 (0.79, 1.41) | 0.72 |  |
| Gender |  |  | 0.38 |  |  | 0.35 |
| Male | 0.78 (0.67, 0.91) | < 0.05 |  | 0.94 (0.81, 1.11) | 0.47 |  |
| Female | 0.93 (0.81, 1.07) | 0.33 |  | 0.99 (0.85, 1.15) | 0.89 |  |
| Residence |  |  | < 0.05 |  |  | 0.67 |
| City | 0.45 (0.18, 1.04) | 0.07 |  | 0.17 (0.48, 2.73) | 0.72 |  |
| Town | 1.06 (0.90, 1.24) | 0.51 |  | 0.97 (0.82, 1.14) | 0.70 |  |
| Rural | 0.77 (0.67, 0.88) | < 0.05 |  | 0.94 (0.81, 1.08) | 0.38 |  |
| BMI (kg/m^2^) |  |  | < 0.05 |  |  | < 0.05 |
| <18.5 | 0.79 (0.63, 0.98) | < 0.05 |  | 0.88 (0.69, 1.11) | 0.27 |  |
| 18.5-23.9 | 0.91 (0.79, 1.04) | 0.17 |  | 0.97 (0.84, 1.12) | 0.68 |  |
| ≥ 24 | 0.90 (0.74, 1.09) | 0.28 |  | 1.09 (0.89, 1.33) | 0.39 |  |
| Smoking status |  |  | 0.20 |  |  | 0.12 |
| No | 0.88 (0.79, 0.98) | < 0.05 |  | 1.00 (0.89, 1.13) | 0.94 |  |
| Yes | 0.76 (0.59, 0.97) | < 0.05 |  | 0.81 (0.63, 1.04) | 0.10 |  |
| Drinking status |  |  | 0.72 |  |  | 0.65 |
| No | 0.86 (0.77, 0.96) | < 0.05 |  | 0.93 (0.83, 1.04) | 0.22 |  |
| Yes | 0.86 (0.67, 1.12) | 0.27 |  | 1.20 (0.92, 1.57) | 0.19 |  |

BMI, body mass index; CI, confidence interval; OR, odds ratio.

ORs and 95 % CIs were adjusted for age (years), gender (men, women), BMI (kg/m^2^), annual income level (≥30,000, <30,000 yuan), ethnicity (Han or others), exercise status (yes, no), labor status (yes, no), marital status (live with spouse, live without spouse), residence (city, town, or rural), smoking status (yes, no), drinking status (yes, no), hypertension (yes, no), diabetes (yes, no), and cardiovascular disease (yes, no).

* indicates *P* for interaction between strata and solids fuel exposure.

**Supplementary Table S4.** Subgroup analyses of DDS and sleep quality and sleep duration.

|  | Sleep quality | | | Sleep duration | | |
| --- | --- | --- | --- | --- | --- | --- |
|  | **OR (95 % CI)** | *P* _value_ | *P* _interaction*_ | **OR (95 % CI)** | *P* _value_ | *P* _interaction*_ |
| Age (years) |  |  | 0.99 |  |  | 0.96 |
| 65-79 | 1.38 (1.19, 1.60) | < 0.05 |  | 1.13 (0.98, 1.31) | 0.10 |  |
| 80-99 | 1.65 (1.44, 1.89) | < 0.05 |  | 1.22 (1.06, 1.41) | < 0.05 |  |
| ≥ 100 | 1.40 (1.11, 1.77) | < 0.05 |  | 1.16 (0.90, 1.50) | 0.24 |  |
| Gender |  |  | 0.32 |  |  | 0.11 |
| Male | 1.44 (1.25, 1.65) | < 0.05 |  | 1.25 (1.08, 1.43) | < 0.05 |  |
| Female | 1.54 (1.36, 1.75) | < 0.05 |  | 1.13 (0.99, 1.29) | 0.07 |  |
| Residence |  |  | < 0.05 |  |  | 0.63 |
| City | 1.37 (1.08, 1.74) | < 0.05 |  | 1.28 (1.01, 1.64) | < 0.05 |  |
| Town | 1.33 (1.14, 1.55) | < 0.05 |  | 1.20 (1.03, 1.40) | < 0.05 |  |
| Rural | 1.71 (1.49, 1.95) | < 0.05 |  | 1.15 (1.00, 1.33) | < 0.05 |  |
| BMI (kg/m^2^) |  |  | < 0.05 |  |  | 0.40 |
| <18.5 | 1.30 (1.06, 1.60) | < 0.05 |  | 1.10 (0.88, 1.37) | 0.41 |  |
| 18.5-23.9 | 1.63 (1.44, 1.86) | < 0.05 |  | 1.26 (1.10, 1.44) | < 0.05 |  |
| ≥ 24 | 1.34 (1.14, 1.58) | < 0.05 |  | 1.10 (0.93, 1.30) | 0.26 |  |
| Smoking status |  |  | 0.63 |  |  | 0.24 |
| No | 1.49 (1.35, 1.65) | < 0.05 |  | 1.22 (1.10, 1.36) | < 0.05 |  |
| Yes | 1.54 (1.23, 1.94) | < 0.05 |  | 1.00 (0.80, 1.26) | 0.99 |  |
| Drinking status |  |  | 0.44 |  |  | 0.07 |
| No | 1.47 (1.33, 1.63) | < 0.05 |  | 1.16 (1.04, 1.28) | < 0.05 |  |
| Yes | 1.67 (1.31, 2.12) | < 0.05 |  | 1.35 (1.05, 1.72) | < 0.05 |  |

BMI, body mass index; CI, confidence interval; DDS, dietary diversity score; OR, odds ratio.

ORs and 95 % CIs were adjusted for age (years), gender (men, women), BMI (kg/m^2^), annual income level (≥30,000, <30,000 yuan), ethnicity (Han or others), exercise status (yes, no), labor status (yes, no), marital status (live with spouse, live without spouse), residence (city, town, or rural), smoking status (yes, no), drinking status (yes, no), hypertension (yes, no), diabetes (yes, no), and cardiovascular disease (yes, no).

* indicates *P* for interaction between strata and DDS.

**Supplementary Table S5.** Subgroup analyses of AIDDS and sleep quality and sleep duration.

|  | Sleep quality | | | Sleep duration | | |
| --- | --- | --- | --- | --- | --- | --- |
|  | **OR (95 % CI)** | *P* _value_ | *P* _interaction*_ | **OR (95 % CI)** | *P* _value_ | *P* _interaction*_ |
| Age (years) |  |  | 0.69 |  |  | 0.98 |
| 65-79 | 1.43 (1.24, 1.65) | < 0.05 |  | 1.21 (1.05, 1.40) | < 0.05 |  |
| 80-99 | 1.69 (1.47, 1.94) | < 0.05 |  | 1.21 (1.04,1.39) | < 0.05 |  |
| ≥ 100 | 1.30 (1.02, 1.67) | < 0.05 |  | 1.26 (0.97, 1.64) | 0.08 |  |
| Gender |  |  | 0.22 |  |  | 0.54 |
| Male | 1.44 (1.26, 1.65) | < 0.05 |  | 1.21 (1.06, 1.39) | < 0.05 |  |
| Female | 1.60 (1.41, 1.82) | < 0.05 |  | 1.21 (1.06, 1.39) | < 0.05 |  |
| Residence |  |  | < 0.05 |  |  | 0.19 |
| City | 1.19 (0.97, 1.45) | 0.09 |  | 1.11 (0.90, 1.35) | 0.33 |  |
| Town | 1.39 (1.19, 1.63) | < 0.05 |  | 1.16 (0.98, 1.36) | 0.08 |  |
| Rural | 1.91 (1.66, 2.20) | < 0.05 |  | 1.34 (1.16, 1.55) | < 0.05 |  |
| BMI (kg/m^2^) |  |  | < 0.05 |  |  | 0.83 |
| <18.5 | 1.49 (1.20, 1.85) | < 0.05 |  | 1.22 (0.97, 1.53) | 0.09 |  |
| 18.5-23.9 | 1.61 (1.41, 1.83) | < 0.05 |  | 1.24 (1.09, 1.41) | < 0.05 |  |
| ≥ 24 | 1.41 (1.20, 1.65) | < 0.05 |  | 1.14 (0.97, 1.34) | 0.10 |  |
| Smoking status |  |  | 0.79 |  |  | 0.11 |
| No | 1.55 (1.40, 1.71) | < 0.05 |  | 1.27 (1.14, 1.41) | < 0.05 |  |
| Yes | 1.48 (1.18, 1.85) | < 0.05 |  | 1.00 (0.80, 1.26) | < 0.05 |  |
| Drinking status |  |  | 0.76 |  |  | < 0.05 |
| No | 1.52 (1.37, 1.68) | < 0.05 |  | 1.17 (1.06, 1.30) | < 0.05 |  |
| Yes | 1.60 (1.26, 2.02) | < 0.05 |  | 1.47 (1.16, 1.86) | < 0.05 |  |

AIDDS, anti-inflammatory dietary diversity score; BMI, body mass index; CI, confidence interval; OR, odds ratio.

ORs and 95 % CIs were adjusted for age (years), gender (men, women), BMI (kg/m^2^), annual income level (≥30,000, <30,000 yuan), ethnicity (Han or others), exercise status (yes, no), labor status (yes, no), marital status (live with spouse, live without spouse), residence (city, town, or rural), smoking status (yes, no), drinking status (yes, no), hypertension (yes, no), diabetes (yes, no), and cardiovascular disease (yes, no).

* indicates *P* for interaction between strata and AIDDS.

**Supplementary Table S6.** Sensitivity analyses: ORs and 95% CIs for the associations of household solids fuel use with sleep quality and sleep duration among the participants with additional adjustment for mold exposure.

| **Variables** | **N _event_/N _total_** | **Model 1** | **Model 2** | **Model 3** |
| --- | --- | --- | --- | --- |
|  |  | **OR (95 % CI)** | **OR (95 % CI)** | **OR (95 % CI)** |
| **Sleep quality** | | | | |
| **Household fuel** |  |  |  |  |
| Clean fuels | 3,533/6,464 | 1.0 (Reference) | 1.0 (Reference) | 1.0 (Reference) |
| Solid fuels | 1,315/2,657 | 0.81 (0.74, 0.89) | 0.81 (0.74, 0.89) | 0.88 (0.80, 0.98) |
| *P* value |  | < 0.05 | < 0.05 | < 0.05 |
| **Sleep duration** |  | | | |
| **Household fuel** |  |  |  |  |
| Clean fuels | 2,410/6,464 | 1.0 (Reference) | 1.0 (Reference) | 1.0 (Reference) |
| Solid fuels | 914/2,657 | 0.88 (0.80, 0.97) | 0.87 (0.79, 0.96) | 0.98 (0.88, 1.09) |
| *P* value |  | < 0.05 | < 0.05 | 0.74 |

CI, confidence interval; OR, odds ratio.

Model 1: Crude model;

Model 2: Adjusted for age (years), gender (men, women), and BMI (kg/m^2^);

Model 3: Further adjusted for annual income level (≥30,000, <30,000 yuan), ethnicity (Han or others), exercise status (yes, no), labor status (yes, no), marital status (live with spouse, live without spouse), residence (city, town, or rural), smoking status (yes, no), drinking status (yes, no), hypertension (yes, no), diabetes (yes, no), cardiovascular disease (yes, no), and mold exposure (yes, or).

**Supplementary Table S7.** Sensitivity analyses: ORs and 95% CIs for the associations of DDS and AIDDS with sleep quality and sleep duration among the participants with additional adjustment for mold exposure.

| **Variables** | **N _event_/N _total_** | **Model 1** | **Model 2** | **Model 3** |
| --- | --- | --- | --- | --- |
|  |  | **OR (95 % CI)** | **OR (95 % CI)** | **OR (95 % CI)** |
| **Sleep quality** | | | | |
| **DDS** | | | | |
| Low | 1,963/4,162 | 1.00 (Reference) | 1.00 (Reference) | 1.00 (Reference) |
| High | 2,885/4,959 | 1.56 (1.43, 1.69) | 1.54 (1.41, 1.68) | 1.47 (1.34, 1.62) |
| *P* value |  | < 0.05 | < 0.05 | < 0.05 |
| Continuous * | 4,848/9,121 | 1.13 (1.11, 1.16) | 1.13 (1.10, 1.15) | 1.12 (1.10, 1.15) |
| **AIDDS** |  | | | |
| Low | 2,535/5,256 | 1.00 (Reference) | 1.00 (Reference) | 1.00 (Reference) |
| High | 2,313/3,865 | 1.60 (1.47, 1.74) | 1.56 (1.43, 1.70) | 1.50 (1.37, 1.65) |
| *P* value |  | < 0.05 | < 0.05 | < 0.05 |
| Continuous * | 4,848/9,121 | 1.25 (1.20, 1.29) | 1.24 (1.19, 1.28) | 1.22 (1.17, 1.27) |
| **Sleep duration** |  | | | |
| **DDS** |  | | | |
| Low | 1,372/4,162 | 1.00 (Reference) | 1.00 (Reference) | 1.00 (Reference) |
| High | 1,952/4,959 | 1.32 (1.21, 1.44) | 1.26 (1.16, 1.38) | 1.17 (1.06, 1.29) |
| *P* value |  | < 0.05 | < 0.05 | < 0.05 |
| Continuous * | 4,848/9,121 | 1.09 (1.07, 1.12) | 1.08 (1.06, 1.10) | 1.06 (1.03, 1.09) |
| **AIDDS** |  | | | |
| Low | 1,750/5,256 | 1.00 (Reference) | 1.00 (Reference) | 1.00 (Reference) |
| High | 1,574/3,865 | 1.38 (1.26, 1.50) | 1.30 (1.18, 1.42) | 1.20 (1.10, 1.32) |
| *P* value |  | < 0.05 | < 0.05 | < 0.05 |
| Continuous * | 3,324/9,121 | 1.17 (1.13, 1.21) | 1.13 (1.09, 1.18) | 1.10 (1.05, 1.14) |

AIDDS, anti-inflammatory dietary diversity score**;** CI, confidence interval; DDS, dietary diversity score; OR, odds ratio.

Model 1: Crude model;

Model 2: Adjusted for age (years), gender (men, women), and BMI (kg/m^2^);

Model 3: Further adjusted for annual income level (≥30,000, <30,000 yuan), ethnicity (Han or others), exercise status (yes, no), labor status (yes, no), marital status (live with spouse, live without spouse), residence (city, town, or rural), smoking status (yes, no), drinking status (yes, no), hypertension (yes, no), diabetes (yes, no), cardiovascular disease (yes, no), and mold exposure (yes, no).

* Continuous were calculated by per one score increase.

**Supplementary Table S8.** Sensitivity analyses: ORs and 95% CIs for the associations of household solid fuel use with sleep quality and sleep duration, after excluding participants who reported respiratory diseases.

| **Variables** | **N _event_/N _total_** | **Model 1** | **Model 2** | **Model 3** |
| --- | --- | --- | --- | --- |
|  |  | **OR (95 % CI)** | **OR (95 % CI)** | **OR (95 % CI)** |
| **Sleep quality** | | | | |
| **Household fuel** |  |  |  |  |
| Clean fuels | 3,206/5,785 | 1.0 (Reference) | 1.0 (Reference) | 1.0 (Reference) |
| Solid fuels | 1,216/2,421 | 0.81 (0.74, 0.89) | 0.81 (0.74, 0.90) | 0.87 (0.78, 0.97) |
| *P* value |  | < 0.05 | < 0.05 | < 0.05 |
| **Sleep duration** | | | | |
| **Household fuel** |  |  |  |  |
| Clean fuels | 2,184/5,785 | 1.0 (Reference) | 1.0 (Reference) | 1.0 (Reference) |
| Solid fuels | 839/2,421 | 0.87 (0.79, 0.97) | 0.87 (0.78, 0.96) | 0.97 (0.86, 1.08) |
| *P* value |  | < 0.05 | < 0.05 | 0.55 |

CI, confidence interval; OR, odds ratio.

Model 1: Crude model;

Model 2: Adjusted for age (years), gender (men, women), and BMI (kg/m^2^);

Model 3: Further adjusted for annual income level (≥30,000, <30,000 yuan), ethnicity (Han or others), exercise status (yes, no), labor status (yes, no), marital status (live with spouse, live without spouse), residence (city, town, or rural), smoking status (yes, no), drinking status (yes, no), hypertension (yes, no), diabetes (yes, no), and cardiovascular disease (yes, no).

**Supplementary Table S9.** Sensitivity analyses: ORs and 95% CIs for the associations of DDS and AIDDS with sleep quality and sleep duration, after excluding participants who reported respiratory diseases.

| **Variables** | **N _event_/N _total_** | **Model 1** | **Model 2** | **Model 3** |
| --- | --- | --- | --- | --- |
|  |  | **OR (95 % CI)** | **OR (95 % CI)** | **OR (95 % CI)** |
| **Sleep quality** | | | | |
| **DDS** | | | | |
| Low | 1,781/3,752 | 1.00 (Reference) | 1.00 (Reference) | 1.00 (Reference) |
| High | 2,641/4,454 | 1.61 (1.48, 1.76) | 1.59 (1.45, 1.74) | 1.53 (1.39, 1.68) |
| *P* value |  | < 0.05 | < 0.05 | < 0.05 |
| Continuous * | 4,422/8,206 | 1.14 (1.12, 1.17) | 1.14 (1.11, 1.16) | 1.13 (1.10, 1.16) |
| **AIDDS** | | | | |
| Low | 2,304/4,724 | 1.00 (Reference) | 1.00 (Reference) | 1.00 (Reference) |
| High | 2,118/3,482 | 1.63 (1.49, 1.78) | 1.58 (1.44, 1.73) | 1.52 (1.38, 1.68) |
| *P* value |  | < 0.05 | < 0.05 | < 0.05 |
| Continuous * | 4,422/8,206 | 1.26 (1.21, 1.31) | 1.25 (1.20, 1.30) | 1.23 (1.18, 1.29) |
| **Sleep duration** | | | | |
| **DDS** | | | | |
| Low | 1,246/4,162 | 1.00 (Reference) | 1.00 (Reference) | 1.00 (Reference) |
| High | 1,777/3,752 | 1.34 (1.22, 1.46) | 1.27 (1.15, 1.39) | 1.18 (1.06, 1.30) |
| *P* value |  | < 0.05 | < 0.05 | < 0.05 |
| Continuous * | 3,023/8,206 | 1.10 (1.08, 1.12) | 1.08 (1.06, 1.11) | 1.06 (1.04, 1.09) |
| **AIDDS** | | | | |
| Low | 1,585/3,482 | 1.00 (Reference) | 1.00 (Reference) | 1.00 (Reference) |
| High | 1,438/4,724 | 1.39 (1.27, 1.53) | 1.30 (1.18, 1.43) | 1.21 (1.11, 1.34) |
| *P* value |  | < 0.05 | < 0.05 | < 0.05 |
| Continuous * | 3,023/8,206 | 1.18 (1.14, 1.23) | 1.14 (1.09, 1.18) | 1.10 (1.06, 1.15) |

AIDDS, anti-inflammatory dietary diversity score**;** CI, confidence interval; DDS, dietary diversity score; OR, odds ratio.

Model 1: Crude model;

Model 2: Adjusted for age (years), gender (men, women), and BMI (kg/m^2^);

Model 3: Further adjusted for annual income level (≥30,000, <30,000 yuan), ethnicity (Han or others), exercise status (yes, no), labor status (yes, no), marital status (live with spouse, live without spouse), residence (city, town, or rural), smoking status (yes, no), drinking status (yes, no), hypertension (yes, no), diabetes (yes, no), and cardiovascular disease (yes, no).

* Continuous were calculated by per one score increase.

**Supplementary Table S10.** Sensitivity analyses: ORs and 95% CIs for the associations of household solid fuel use with sleep duration, after using a more inclusive sleep duration range (6-9 hours).

| **Variables** | **N _event_/N _total_** | **Model 1** | **Model 2** | **Model 3** |
| --- | --- | --- | --- | --- |
|  |  | **OR (95 % CI)** | **OR (95 % CI)** | **OR (95 % CI)** |
| **Sleep duration** | | | | |
| **Household fuel** |  |  |  |  |
| Clean fuels | 4,034/6,464 | 1.0 (Reference) | 1.0 (Reference) | 1.0 (Reference) |
| Solid fuels | 1,603/2,657 | 0.92 (0.84, 1.01) | 0.90 (0.82, 0.99) | 1.03 (0.93, 1.14) |
| *P* value |  | 0.06 | < 0.05 | 0.61 |

CI, confidence interval; OR, odds ratio.

Model 1: Crude model;

Model 2: Adjusted for age (years), gender (men, women), and BMI (kg/m^2^);

Model 3: Further adjusted for annual income level (≥30,000, <30,000 yuan), ethnicity (Han or others), exercise status (yes, no), labor status (yes, no), marital status (live with spouse, live without spouse), residence (city, town, or rural), smoking status (yes, no), drinking status (yes, no), hypertension (yes, no), diabetes (yes, no), and cardiovascular disease (yes, no).

**Supplementary Table S11.** Sensitivity analyses: ORs and 95% CIs for the associations of DDS and AIDDS with sleep duration, after using a more inclusive sleep duration range (6–9 hours).

| **Variables** | **N _event_/N _total_** | **Model 1** | **Model 2** | **Model 3** |
| --- | --- | --- | --- | --- |
|  |  | **OR (95 % CI)** | **OR (95 % CI)** | **OR (95 % CI)** |
| **Sleep duration** | | | | |
| **DDS** | | | | |
| Low | 2,390/4,162 | 1.00 (Reference) | 1.00 (Reference) | 1.00 (Reference) |
| High | 3,247/4,959 | 1.41 (1.29, 1.53) | 1.32 (1.21, 1.45) | 1.23 (1.12, 1.35) |
| *P* value |  | < 0.05 | < 0.05 | < 0.05 |
| Continuous * | 5,637/9,121 | 1.11 (1.09, 1.14) | 1.10 (1.07, 1.12) | 1.08 (1.05, 1.10) |
| **AIDDS** | | | | |
| Low | 3,046/5,256 | 1.00 (Reference) | 1.00 (Reference) | 1.00 (Reference) |
| High | 2,591/3,865 | 1.48 (1.35, 1.61) | 1.35 (1.23, 1.48) | 1.26 (1.15, 1.39) |
| *P* value |  | < 0.05 | < 0.05 | < 0.05 |
| Continuous * | 5,637/9,121 | 1.20 (1.16, 1.25) | 1.15 (1.11, 1.20) | 1.11 (1.07, 1.16) |

AIDDS, anti-inflammatory dietary diversity score**;** CI, confidence interval; DDS, dietary diversity score; OR, odds ratio.

Model 1: Crude model;

Model 2: Adjusted for age (years), gender (men, women), and BMI (kg/m^2^);

Model 3: Further adjusted for annual income level (≥30,000, <30,000 yuan), ethnicity (Han or others), exercise status (yes, no), labor status (yes, no), marital status (live with spouse, live without spouse), residence (city, town, or rural), smoking status (yes, no), drinking status (yes, no), hypertension (yes, no), diabetes (yes, no), and cardiovascular disease (yes, no).

* Continuous were calculated by per one score increase.

**Supplementary Table S12.** Sensitivity analyses: E-values for the associations between household solid fuel use, dietary diversity, and sleep quality and sleep duration.

| Outcomes | Exposure | E-values* for OR (and for CI) |
| --- | --- | --- |
| Sleep quality | Solid fuels vs. Clean fuels | 1.59 (1.28) |
|  | High DDS vs. Low DDS | 2.37 (2.08) |
|  | High AIDDS vs. Low AIDDS | 2.43 (2.13) |
| Sleep duration | Solid fuels vs. Clean fuels | 1.21 (1.00) |
|  | High DDS vs. Low DDS | 1.64 (1.34) |
|  | High AIDDS vs. Low AIDDS | 1.74 (1.46) |

AIDDS, anti-inflammatory dietary diversity score; CI, confidence interval; DDS, dietary diversity score; OR, odds ratio.

* E-value represents the minimum strength of association needed between an unmeasured confounder and both the exposure and the outcome to fully explain away the exposure-outcome association.
